# Vitamin D levels in patients with recurrent aphthous stomatitis

**DOI:** 10.1186/s12903-018-0653-9

**Published:** 2018-11-09

**Authors:** Aynure Öztekin, Coşkun Öztekin

**Affiliations:** 1 Department of Dermatology, Hitit University Medical School, Çorum, Turkey; 2 Department of Family Medicine, Hitit University Medical School, Çorum, Turkey

## Abstract

**Background:**

Lower serum vitamin D levels, a major public health problem worldwide, has been found to be associated with various infectious diseases, cancers, autoimmune and dermatological diseases**.** The serum levels of vitamin D in patients with recurrent aphthous stomatitis are not clear. We investigated the vitamin D levels in patients with recurrent aphthous stomatitis.

**Methods:**

Forty patients with recurrent aphthous stomatitis (Group I) and 70 healthy controls (Group II) included in the study. The characteristics of aphthous lesions (duration of disease and remission, frequency, diameter and number of the lesions) and demographics of the participants were recorded. Serum 25-hydroxycholecalciferol levels were measured using electrochemiluminescence binding method.

**Results:**

There was no statistically significant difference between the groups in terms of age (*p* = 0.06) and sex (*p* = 0.4). Other baseline characteristics were not significantly different between the groups (*p* > 0.05 for all). The mean diameter of aphthous lesions was 0.5 (0.4–0.6) cm and the mean number of lesions was 2.2 ± 1.5. Serum vitamin D levels were 11 ± 7.04 ng/ml in Group I and 16.4 ± 10.19 ng/ml in Group II. Serum vitamin D levels were significantly lower in patients with recurrent aphthous stomatitis (*p* = 0.004).

**Conclusions:**

The present study showed lower vitamin D levels in patients with recurrent aphthous stomatitis compared to healthy controls.

## Introduction

Aphthous stomatitis is the painful ulcers of the oral mucous membranes. These idiopathic noninfectious lesions are characterized by recurrent painful attacks (commonly known as “canker sores”). Aphthous stomatitis affects approximately 20% of the general population [1]. Risk factors include local trauma, emotional or physiologic stress, allergy, toxin exposure, vitamin deficiency, poor oral hygiene, menstruation, and alterations in the oral flora [1].

Recent studies investigated the role of several vitamins including vitamin B1, B2, B6, B12 and folic acid in the etiopathogenesis of recurrent aphthous stomatitis [2–5]. Nolan et al. [2] found that 28% of 60 patients with recurrent oral ulcers had a deficiency in at least one of the B1, B2, and B6 vitamins. Replacement therapy in these deficient individuals with recurrent oral ulcers displayed a significant improvement within 1 month. Lalla et al. [6] evaluated the effect of daily multivitamin supplementation (vitamins A, B1, B2, B3, B5, B6, B9, B12, C, D, and E) on the number and duration of recurrent aphthous stomatitis episodes but found no significant improvement in the number or duration of episodes. In contrast, Petersen et al. [7, 8] reported significant improvement in the number of recurrent aphthous lesions using combination vitamin/herbal supplement even in the absence of serologic vitamin deficiency.

Vitamin D is a fat-soluble secosteroid and functions primarily in the regulation of the calcium and phosphorus balance [9]. Recent studies over the past years have revealed a broader role of vitamin D; not only in skeletal and cardiovascular disorders but also in cancers, central nervous system diseases, infections, autoimmune, and dermatological disorders [10]. It is known that the risk factors for the development of autoimmune diseases are a mosaic, which includes family history, genetic predisposition, hormonal status and environmental features. In accordance, lower vitamin D levels and vitamin D receptor polymorphism has been suggested as important risk factors for the development of autoimmune diseases [11]. Previous studies indicate that innate and acquired immunity play an important role in the development of recurrent aphthous stomatitis [12].

The role of vitamin D deficiency in patients with recurrent aphthous stomatitis is not well known. The aim of the present study is to investigate the serum levels of vitamin D in patients with recurrent aphthous stomatitis.

## Methods

This cross-sectional study complied with the tenets of the Declaration of Helsinki and was approved by the Ethical Committee of Atatürk University, Erzurum, Turkey. Written informed consent was obtained from all the participants before the study. This study was conducted in the Dermatology Clinic of Palandöken State Hospital, Erzurum, Turkey.

A total of 40 patients with recurrent aphthous stomatitis (Group I) and 70 healthy controls (Group II) were included in the study. Participants were 18 years and older with a validated history of at least three episodes of idiopathic recurrent aphthous stomatitis within the previous 12 months. Only minor aphthous lesions < 1 cm in diameter were included in the study. One experienced investigator evaluated participants about the location, duration, frequency, diameter, number, and appearance of aphthous lesions. Exclusion criteria were as follows: (1) age < 18 years and > 50 years, (2) presence of systemic disease that can cause oral ulceration, (3) pregnancy and lactation (4) use of vitamin D supplementation within last 6 months. Participants in the control group consisted of healthy volunteers who had no complaints and systemic disease.

Age, gender, marital status, level of education, place of residence (rural or urban), smoking and alcohol use, and drug use characteristics of all participants were recorded. Diagnosis of recurrent aphthous stomatitis was based on physical examinations by an experienced dermatologist and patient history.

### Serum collection

Blood from the forearm vein was collected into 5-ml Vacutainer tubes with no anticoagulant. The blood samples were centrifuged (1000×g, 15 min, 4 °C) to separate serum. Serum was removed and immediately stored at − 80 °C until analyzed.

### Biochemical measurements

Serum 25-hydroxycholecalciferol measurements were performed using electrochemiluminescence binding method (COBAS reagent kit; COBAS e601 analyzer series, Roche Diagnostics, Basel, Switzerland). The results were expressed in ng/dl.

### Statistical analysis

All data were entered into a spreadsheet, and statistical analyses were performed using R 3.3.2v (open source). Data are shown as mean ± standard deviation for continuous variables, as median (minimum-maximum) for ordinal variables, and as frequency with percent for categorical variables. To evaluate the level of data normality for continuous variables, the Kolmogorov Smirnov test was used. Categorical comparisons were made by chi-square test. Independent sample t-test was used for comparing the means of continuous variables.

For more than two independent groups, the Kruskal Wallis test was used for non-normally distributed variables. A Spearman’s rho correlation was used to analyze the association between non-normally distributed variables. Correlations between normally distributed variables were analyzed by Pearson’s correlation coefficient. A *p* value of < 0.05 was considered statistically significant.

## Results

A total of 40 patients (25 females, 15 males) were included in Group I and 70 healthy individuals in Group II (38 females and 32 males). The mean age of the participants in Group I was 31.2 ± 10.05 years and in Groups II was 27.44 ± 7.96 years. There was no statistically significant difference between the groups in terms of age (*p* = 0.06) and gender (*p* = 0.4). Other baseline characteristics including marital status, level of education, and place of residence were not significantly different between the groups (*p* > 0.05 for all). Table 1 summarizes the demographics and baseline characteristics of the groups in detail.

**Table 1:** Demographics and baseline characteristics of the groups

	Groups		*P value*
	Group I	Control	
Age (years)	31.20 ± 10.05	27.44 ± 7.96	0.06
Sex			
Male	15 (37.5)	32 (45.71)	0.4
Female	25 (62.5)	38 (54.29)	
Marital status			
Married	26 (65)	34 (48.6)	0.1
Single	14 (35)	36 (51.4)	
Level of education			
Primary school	14 (35)	16 (22.86)	0.07
Secondary school	7 (17.5)	4 (5.71)	
High school	9 (22.5)	23 (32.86)	
University	10 (25)	27 (38.57)	
Place of residence			
Rural	5 (12.5)	7 (10)	0.76
Urban	35 (87.5)	63 (90)	

One patient (2.5%) was smoking and none of the patients (1.82%) were using alcohol in Group I. Ten patients (25%) had a history of drug use in Group I. Twenty-one patients (52.5%) had a family history of aphthous stomatitis.

The mean duration of recurrent aphthous stomatitis was 42 (24–65) months. The mean diameter of the lesions was 0.5 (0.4–0.6) cm. The mean number of the lesions per patient was 2 (1–7). The mean frequency of recurrence was 15 (7–90) days. The mean healing time was 8.2 ± 3.38 days. The mean duration of remission was 7 (5–14) months. There was no significant correlation between serum vitamin D levels and the diameter of the aphthous lesions (*r* = 0.044, *p* = 0.79), the numbers of lesions (*r* = 0.074, *p* = 0.651), and the mean healing time (*r* = 0.013, *p* = 0.935).

**Fig. 1:**
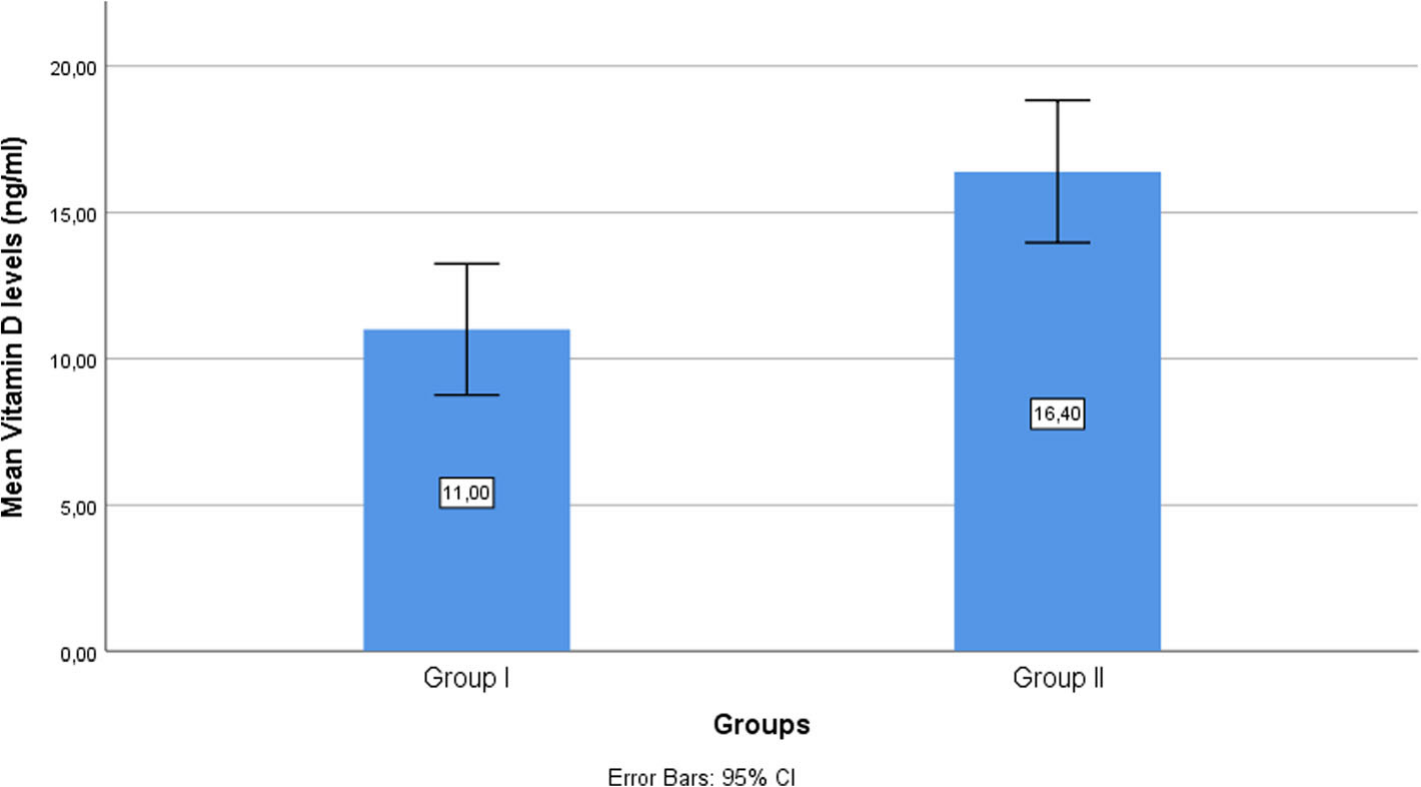
The mean serum vitamin D concentrations of the groups in an error bar graph

Serum vitamin D levels were 11.00 ± 7.03 ng/ml in Group I and 16.40 ± 10.19 ng/ml in Group II. There was a statistically significant difference between the groups in terms of serum vitamin D levels (*p* = 0.004). Male patients with recurrent aphthous stomatitis had a higher serum vitamin D levels compared to female patients (15.27 ± 6.94 versus 8.44 ± 5.82) in Group I (*p* = 0.002). Figure 1 shows the mean serum vitamin D concentrations of the groups in an error bar graph.

## Discussion

This study evaluated the vitamin D levels in the serum of patients with recurrent aphthous stomatitis. We found decreased levels of vitamin D in the serum of patients with recurrent aphthous stomatitis compared to healthy control subjects.

Since the exact etiology of recurrent aphthous stomatitis is unknown, there is no effective and curative therapy. Current treatment options (topical analgesics, antiseptics, corticosteroids and systemic therapy) are nonspecific and have limited efficacy. The role of multivitamin supplementation including vitamin D in the treatment of recurrent aphthous stomatitis is controversial and published studies reported conflicting results [6–8]. Children with periodic fever, aphthous stomatitis, pharyngitis, and cervical adenitis (PFAPA) syndrome have decreased levels of vitamin D and replacement treatment seems to significantly reduce the typical PFAPA episodes and their duration [13, 14]. Further studies are necessary to confirm the role of vitamin D in the treatment of recurrent aphthous stomatitis.

The major source of vitamin D in humans is endogenous synthesis in the skin with the help of sunshine. Salmon, mackerel, caviar, and eggs are the other sources of vitamin D [15]. Vitamin D deficiency/insufficiency at all ages is a major public health problem worldwide [16]. It is not only related with rickets but also with a wide variety of diseases including neoplastic, cardiovascular and autoimmune diseases. There are only several preliminary studies present in the literature evaluating vitamin D levels in patients with aphthous stomatitis. Khabbazi et al. [17], compared the serum 25-hydroxy vitamin D levels in idiopathic minor recurrent aphthous stomatitis patients with age and sex-matched healthy controls. They found that 25-hydroxy vitamin D levels were significantly lower in the study group (12.1 ng/dl vs 27.4 ng/dl). In consistent with this study, we found lower levels of serum 25-hydroxy vitamin D in patients with recurrent aphthous stomatitis compared to healthy individuals with similar age and gender distribution. Another study by Krawiecka et al. [18], reported no significant difference between patients with recurrent aphthous stomatitis and healthy individuals in terms of serum vitamin D levels. Decreased vitamin D levels have been reported in various several disorders, including multiple sclerosis, infectious diseases, diabetes mellitus, systemic lupus erythematosus, rheumatoid arthritis, inflammatory bowel disease, thyroiditis and autoimmune gastritis [19–22]. We found significantly lower levels of vitamin D in patients with recurrent aphthous stomatitis. However, it is not clear whether vitamin D deficiency is the cause or rather a consequence of the disease.

Another finding of the present study is that male patients have higher serum vitamin D levels compared to female patients with recurrent aphthous stomatitis. There are conflicting results among the studies regarding vitamin D level differences by gender in the literature [23, 24]. Al-Horani et al. [23], found the serum vitamin D concentrations in young males and females were 25.82 ng/ml and 21.95 ng/mL, respectively. However, in a longitudinal study by Khosravi-Boroujeni [23] reported slightly higher vitamin D levels and lower vitamin D deficiency in female participants compared to males.

Altunsoy et al. [25], reported the serum levels of 25 (OH) vitamin D in healthy adults with a mean age of 37.58 years (23 male, 17 female) as 22.31 ± 8.8 ng/ml. Another study found that the median value of 25 (OH) vitamin D in 50 healthy adults with a mean age of 53.44 ± 8.4 22 years as 22 (6–40) ng/ml [26]. In the present study, we have found a relatively lower levels of 25 (OH) vitamin D levels compared to previous studies in our healthy control group (16.40 ± 10.19 ng/ml). It is well known that measurement of 25 (OH) vitamin D levels is the best indicator of the vitamin D status in human. The measurement and standardization of 25 (OH) vitamin D levels remains challenging for various reasons. These reasons may include patient related factor (e.g. ethnicity, age, gender), methodological factors (e.g. chemiluminescent immunoassay, high-performance liquid chromatography, radioimmunoassay), and others (e.g. season, geographic location) [27–29]. For example; the correlation between two automated assays in terms of the measurements of serum vitamin D levels was found to be fair and these two methods (the Cobas® Total Vitamin D Assay and the Liaison XL® Total Vitamin D Assay) showed significant bias [28]. Therefore, it would be more meaningful to compare the results of the study within itself until a universal standardization of vitamin D level was established according to factors mentioned before.

## Conclusions

This article provides a significant contribution to the literature regarding levels of vitamin D in patients with recurrent aphthous stomatitis. With our findings, and considering that Vitamin D replacement will not cause significant adverse effects, we recommend vitamin D as supportive treatment in patients with in recurrent aphthous stomatitis. Further studies with larger sample size are necessary to confirm our results and prospective randomized clinical trials will reveal the potential protective/therapeutic role of vitamin D in recurrent aphthous stomatitis.

## Abbreviation

PFAPA: Periodic fever, aphthous stomatitis, pharyngitis, and cervical adenitis
